# Case report: Neoadjuvant systemic therapy for melanoma

**DOI:** 10.1016/j.amsu.2020.05.013

**Published:** 2020-05-25

**Authors:** Natiya Gabuniya, Ankur Khajuria, Jenny L.C. Geh, Daniah Alsaadi

**Affiliations:** a Guy's and St Thomas NHS Foundation Trust, United Kingdom; b Imperial College London, United Kingdom; c University College London, United Kingdom

**Keywords:** Case report, Advanced metastatic melanoma, Neoadjuvant systemic therapy, Skin cancer

## Abstract

We report a case of rapidly enlarging metastatic melanoma in 45-year-old White male following primary resection of thin melanoma five years ago. Location and large size of the lesion possessed significant risk of complications from surgery, therefore provided a challenge in treatment options. Neoadjuvant targeted chemotherapy was commenced and resulted in a significant reduction in size of the lesion, which allowed subsequent safe surgical resection with no residual disease on histopathology results. This case provides a good example of successful utilization of neoadjuvant systemic therapy in advanced metastatic melanoma.

## Highlights

• Outline of advanced metastatic melanoma presentation.
• Successful treatment with neoadjuvant therapy that aided surgical resection and achieved disease free results on follow up.
• Overall improved survival and remission of patient with advanced disease.

## 1 Introduction

Melanoma accounts for 4% of all new cancer cases and is the 5th most common cancer in the UK. Incidence rates are increasing in most European countries and around the world. Prognosis for high-risk melanomas remains poor. Although surgical resection remains a key treatment for stages I-IIIb melanomas, it doesn't improve survival rates alone and has a minimal role in treating advanced regional and distant metastatic disease. The therapeutic value of completion lymphadenectomy following a positive sentinel lymph node biopsy (SLNB) currently remains questionable. New guidance has been published to allow systemic therapy or observation with close follow up following positive sentinel node biopsy (Melanoma focus and JPRAS publication Peach et al.). Sentinel node biopsy is the most important staging tool for stage IB to II patients. The management of advanced melanoma has improved significantly following introduction of systemic therapies.

Neoadjuvant therapy has shown to aid surgical resection and control disease where tumors were previously inoperable. Effectiveness of treatment can be assessed preoperatively by monitoring tumor response and postoperatively by examining resected tissue sample. At the moment neoadjuvant therapy for high-risk melanomas can be considered for irresectable disease, but lately advances of immunotherapy are being incorporated to neoadjuvant phase I and II trials [1]. This clinical case report provides a good example of successful resection and outcome following neoadjuvant chemotherapy.

## 2 Methods

Retrospective overview of clinical case of one patient with rapidly enlarging advanced metastatic melanoma that had a successful outcome following neoadjuvant chemotherapy and subsequent surgical resection. This clinical case is compliant and reported in line with SCARE 2018 criteria [2].

## 3 Case report

In October 2014, a 46-year-old White male presented to clinic with recent history of rapidly growing, painful swelling in left axilla. He was referred by his GP to Melanoma clinic in view that in February 2010 he had a wide local excision of malignant melanoma on his right upper back with 0.72mm Breslow thickness. Swelling in the axilla has been present for 3 months, and he first noticed it during game of tennis. On presentation the patient was systemically well and had no co-morbidities, no other past medical history and no family history of melanoma, however he reported occasional prolonged sun exposure during holidays and mild sun burns in the past. He is a non-smoker and alcohol consumption was mild.

On examination he had a 10 cm firm swelling under left axilla with no obvious surface skin changes. There was no other palpable lymphadenopathy.

**Fig. 1:**
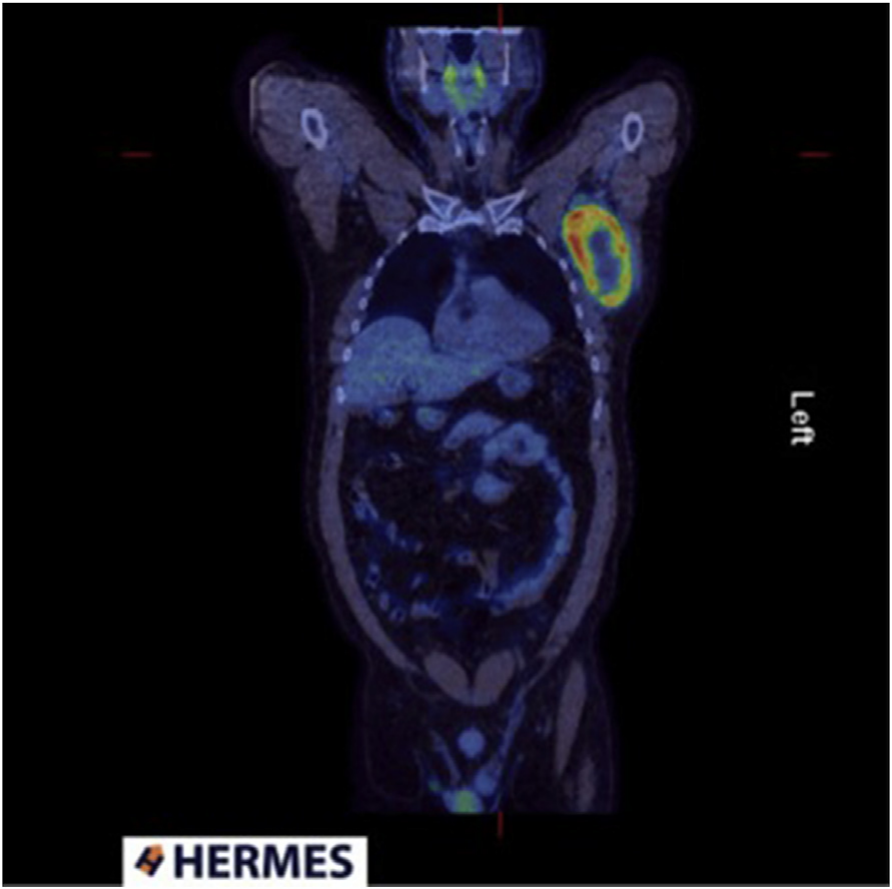
Pre-operative PET scan in 2014.

An ultrasound and fine needle aspiration (FNA) of the mass, confirmed metastatic melanoma with a BRAF V600E mutation. He underwent further scanning to identify any other lesions. Results of brain MRI were normal and PET-CT showed a large FDG avid axillary mass (Fig. 1) likely to represent a lymph node with no metabolically active primary lesion.

Given the size and location of the mass, it was not amenable for safe surgical resection without significant risk of morbidity. It was decided to commence systemic treatment with the BRAF inhibitor, Dabrafenib.

Four month following commencement of systemic therapy there was a significant reduction in size of the lesion that made it suitable and safe for surgical resection. Patient was scheduled on the elective list for axillary node clearance and was operated by Consultant Plastic surgeon, procedure was uncomplicated and patient was discharged home the following day. Specimen was sent to histopathology laboratory and subsequently been reported as no malignancy. This was a complete response to Dabrafenib. It was decided to continue with Dabrafenib to complete a full year of therapy.

**Fig. 2:**
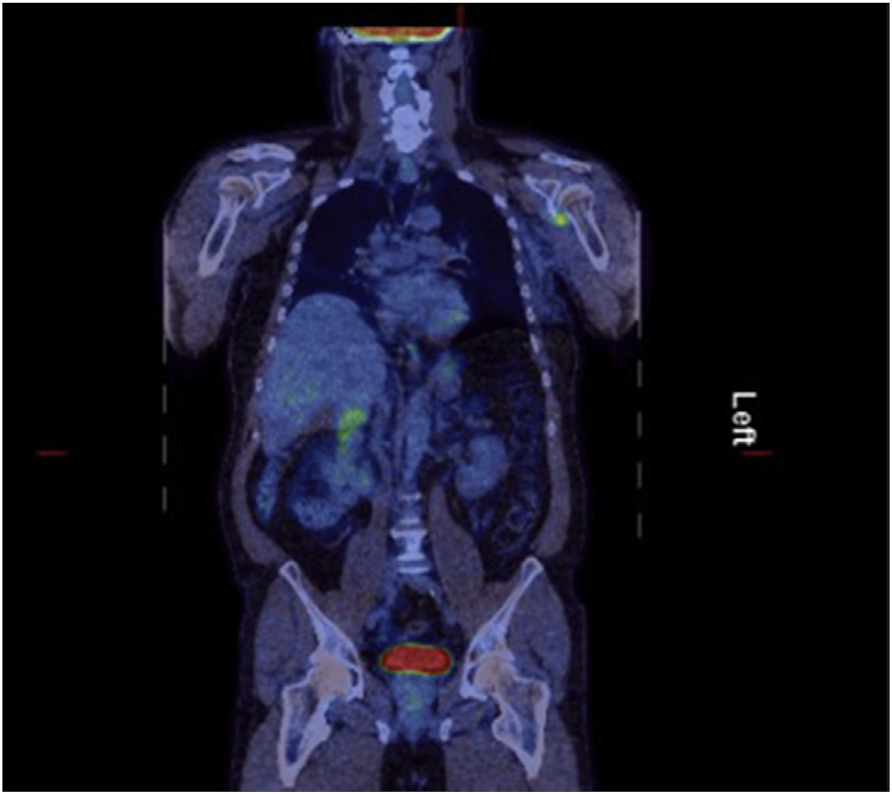
Post-operative scan PET scan in 2015.

**Fig. 3:**
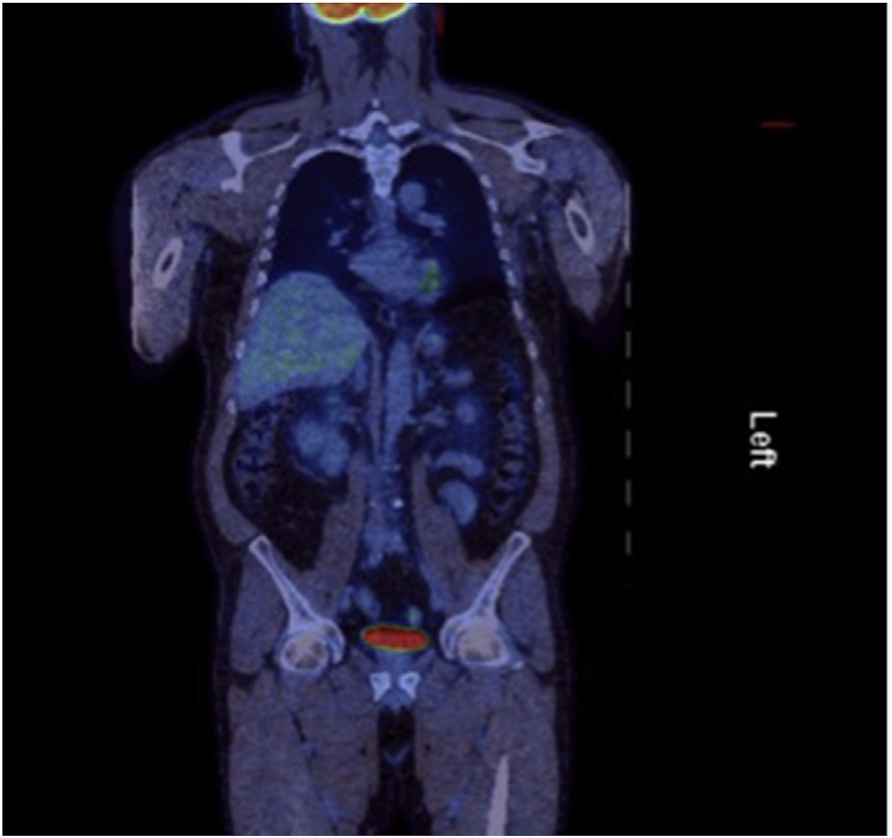
Follow-up post-operative PET scan in 2016.

Patient was regularly followed up in Oncology and Plastic surgery clinics and had yearly PET-CT scans (Fig. 2, Fig. 3) that showed no metastatic disease. Overall recovery was smooth and uncomplicated.

Following 2 years of remission, the patient re-presented with a palpable infraclavicular lymph node. Lesion was small and amenable for surgical excision, following surgical procedure patient completed a course of radiotherapy.

Currently, the patient is completing systemic therapy with Pembrolizumab without any toxic side effects. He continues to be in remission with negative follow up scans.

## 4 Discussion

Neoadjuvant therapy has proven to aid surgical resection of tumors and provide better local control. It helps to adopt more specific therapy approach by monitoring the response of malignancy to therapy pre and post operatively.

Recently established treatments for advanced and metastatic melanoma with BRAF V600 E targeted therapy and CTLA-4 immunotherapy showed dramatic and favorable results [3,4]. The current trials are ongoing.

There are several neoadjuvant therapy trials being conducted that include immunotherapy, anti-CTLA-4 antibody, BRAF and MEK inhibitors, anti-PD1 antibodies and tamilogene laherparepvec (T-VEC).

REDuCTOR trial phase II in Netherlands, is a single center trial that evaluated the outcome of neoadjuvant therapy in patients with primarily unresectable BRAF positive advanced melanoma to aid surgical resection [5]. This approach is different as reduction in tumor size is necessary for resection. Primary outcome measure of this trial includes number of patient in whom resection was made possible by the therapy.

Combi-Neo is a randomized control trial that is comparing dabrafenib and trametinib therapy prior surgery versus surgical resection alone in BRAF mutation positive stage III and IV melanoma. (NCT02231775) [6]. Phase II of this trial will evaluate outcome of 1 year relapse free survival.

Neo Trio study in Melanoma Institute of Australia is evaluating different therapy combinations with dabrafenib, trametinib and pembrolizumab in BRAF positive stage III melanoma (NCT 02858921) [7]. This aims to evaluate whether 3-drug combination provides better reduction in size of the advance melanoma tumor and prevents recurrence post operatively. Outcome of the trial is not yet published.

At the moment there are few published data on survival rates of patients managed with systemic therapy and surgery but reports are promising.

Response to neoadjuvant chemotherapy may identify responder populations in stage III or oligometastatic stage IV melanoma that may benefit from surgery, and who previously may have been palliated, additionally it can potentially spare surgery for advanced non-responders. Surgical resection following neoadjuvant systemic therapy can serve in evaluating the histological responsiveness to therapy. This can help individualize systemic regimens for patients based on their individual response.

Our case provides a good example of favorable outcome of neoadjuvant systemic therapy metastatic melanoma. We have since seen a number of other patients in whom bulky or highly co-morbid resection has been downgraded to a lower risk surgical procedure with the aid of neo-adjuvant systemic therapies.

## 5 Conclusion

In the last decade establishment of systemic therapies has dramatically improved survival rates of advanced melanoma. Although surgery remains a cornerstone of treatment, recent trials and early reports of neoadjuvant and adjuvant therapies has proven to play a crucial role in the management of advanced regional and metastatic melanoma. Neoadjuvant chemotherapy can aid respectability of melanomas to improve local control and decrease surgical morbidity. Surgery continues to have a useful role in controlling disease in both early and late stages of melanoma. Surgical resection following neoadjuvant systemic therapy can serve in evaluating the histological responsiveness to therapy. In the future this can help individualize systemic regimens for patients based on their individual response and improve treatment outcomes.

## Provenance and peer review

Not commissioned, externally peer reviewed.

## Ethical approval

Not applicable – not a research.

## Source of funding

Funding is available from 10.13039/501100000761Imperial College London

## Author contribution

Dr Natiya Gabuniya – data and results analysis, writing case report, report concept and design.

Mr. Ankur Khajuria, Ms. Jenny Geh, Dr Mark Harris – data collection, data analysis.

Dr Daniah Alsaadi – help in organizing the report.

## Registration of research studies

1. Name of the registry
2. Unique Identifying number or registration ID
3. Hyperlink to the registration (must be publicly accessible)

## Guarantor

Dr Natiya Gabuniya.

Ms Jenny Geh.

Mr Ankur Khajuria.

## Declaration of competing interest

Nothing to disclose.
